# Rosmarinic Acid, a New Polyphenol from *Baccaurea ramiflora* Lour. Leaf: A Probable Compound for Its Anti-Inflammatory Activity

**DOI:** 10.3390/antiox3040830

**Published:** 2014-12-03

**Authors:** Talambedu Usha, Sushil Kumar Middha, Malay Bhattacharya, Prakash Lokesh, Arvind Kumar Goyal

**Affiliations:** 1 Department of Biotechnology, Maharani Lakshmi Ammanni College for Women, Bangalore-560012, India; E-Mails: ushatalambedu@gmail.com (T.U.); sushil.middha@gmail.com (S.K.M.); 2 Department of Botany, Kalimpong College, Kalimpong-734301, India; E-Mail: malaykpgc@gmail.com; 3 Padamshree Institute of Management and Science, Bangalore-560060, India; E-Mail: connectloki@gmail.com; 4 Bamboo Technology, Department of Biotechnology, Bodoland University, Kokrajhar-783370, India

**Keywords:** *Baccaurea ramiflora*, rosmarinic acid, anti-inflammatory activity, cytokines

## Abstract

Despite several pharmacological applications of *Baccaurea ramiflora* Lour., studies on the influence of its polyphenol content on pharmacological activity such as anti-inflammatory properties have been scarce. Here we evaluated *in vitro* antioxidant activity, poyphenolics by HPLC and the anti-inflammatory potential of the methanolic leaf extract of *Baccaurea ramiflora* (BME) and its protective effects in carrageenan-induced paw edema model of inflammation in rats. The BME extract contained 79.06 ± 0.03 mg gallic acid equivalent (GAE)/g total polyphenols, 28.80 ± 0.01 mg quercetin equivalent (QE)/g flavonoid and 29.42 ± 0.01 μg cathechin equivalent/g proanthocyanidin respectively and rosmarinic acid (8 mg/kg) as a main component was identified by HPLC. Results demonstrate that administration of BME at the dose of 200 mg/kg can reduce paw edema by over 63%, and it exhibits a dose-response effect. Depending on concentration, the extract exerted scavenging activity on DPPH radical (IC_50_ 36.4 μg/mL), significantly inhibited IL-1β (4.4 pg/mg protein) and TNF-α (0.21 ng/μg protein). Therefore, we conclude BME causes a substantial reduction of inflammation in *in vivo* models. We propose that rosmarinic acid and similar phenolic compounds may be useful in the therapy of inflammation-related injuries.

## 1. Introduction

*Baccaurea ramiflora* Lour. syn. *B. sapida* (Roxb.) Muell. Arg. popularly known as Burmese grapes belongs to the family Euphorbiaceae and is native to Southeast Asia. It is mainly encountered in the sub-Himalayan tract, from Nepal to Sikkim, the Darjeeling hills, Arunachal Pradesh, Tripura, Assam, Bhutan, Burma, Penninsular Malaysia, Tibet and the Andaman islands [1]. It is a slow growing semi-evergreen tree reaching a height of about 25 m [2]. Fruit is 2–3 cm in diameter and is yellowish to red in color with leathery pericarp, three seeded arills embedded in pinkish white pulp [1]. It is variously named in different languages like Mafia in Thai, Latkan in Hindi, Leteku in Assamese, Bhupi in Bengali, *etc.* [2]. The whole plant is traditionally used in Chinese Dai medicine [3]. In Bangladesh, young leaves are used as a vegetable, flavoring agent with curries and minced meat [4]. In India, juice is used orally for constipation, proven to have antioxidant properties [1]. The antiviral and antioxidant properties of the fruit and diuretic activity of stem bark have been reported [1,4,5]. So far fourteen compounds have been isolated from the leaves by various workers [6,7]. A review of the literature showed that though works on hypoglycemic and hypolipidemic activity of *B. ramiflora* leaf have been reported [8], its anti-inflammatory activity is yet to be explored. Keeping this in mind the present study was designed to evaluate *in vivo* anti-inflammatory, *in vitro* antioxidant activity and HPLC analysis of the methanolic extract of the leaf of *B. ramiflora* (BME).

## 2. Experimental Section

### 2.1. Chemicals and Reagents

Rosmarinic acid (HPLC grade), carrageenan were obtained from Sigma (St. Louis, MO, USA), 2,2-diphenyl-1-picryl-hydrazyl (DPPH), quercetin, sodium nitrite (NaNO_2_), trichloroacetic acid (TCA), ascorbic acid, ferric chloride (FeCl_3_), gallic acid were obtained from HiMedia Laboratories Pvt. Ltd., Mumbai, India. Potassium di-hydrogen phosphate (KH_2_PO_4_), di-potassium hydrogen phosphate (K_2_HPO_4_), sodium hydroxide (NaOH), ammonium molybdate, sulfuric acid, potassium ferricyanide (K_2_Fe(CN)_6_), sodium carbonate (Na_2_CO_3_), hydrogen peroxide (H_2_O_2_) and methanol were procured from Merck, Mumbai, India. Diclofenac sodium from Cipla, Mumbai, India and Folin-Ciocalteu (FC) reagent from Sisco Research Laboratory, Mumbai, India. Aluminum chloride (AlCl_3_), Greiss reagent was obtained from Sd fine Chemicals Ltd., Mumbai, India. All chemicals and solvents were of analytical grade.

### 2.2. Plant Material Collection and Extraction

Fresh leaves of *B. ramiflora* were collected from Jalpaiguri, West Bengal, India. The plant material was authenticated by a plant taxonomist and a voucher specimen (Voucher No. KPGC/MB/74) was deposited at Kalimpong Government College, Kalimpong, West Bengal, India.

The leaves were air dried and powdered using a mechanical grinder. 10 g of the powdered leaf was extracted in a Soxhlet apparatus using 80% aqueous methanol (the ratio of plant material to solvent was 1:15 w/v) [9]. The extraction was carried out at boiling temperature for 6 h. The extract obtained was evaporated under pressure at 50 °C to a constant weight and stored at 4 °C until required. The extract was dissolved in double-distilled water (DDW) and dimethyl sulphoxide (DMSO) in the desired concentrations according to the protocols just before use.

### 2.3. In vitro Assay

#### Determination of Biochemical Constituents

The total soluble phenolics (TPC) was determined by the Singleton and Rossi [10] method with a slight modification [11]. Briefly the leaf extract (0.5 mL) was mixed with 0.5 mL of FC reagent (previously diluted to 1:1 ratio with double distilled water) and incubated for 5 min at room temperature (RT), then 1 mL of 2% Na_2_CO_3_ solution was added. After incubation at RT for 10 min, the absorbance of the blue color that developed was read at 730 nm using a Themo UV1-Vis spectrophotometer (Thermo Electron Corporation, England, UK). Gallic acid was used as a standard. The concentration of total phenolic compounds was determined in μg of gallic acid equivalent using an equation obtained from the standard gallic acid graph. The total flavonoid content (TFC) was determined according to Zhishen *et al.* [12] using quercetin as a standard. The plant extract (0.25 mL) was added to 1.25 mL DDW followed by 75 μL of 5% NaNO_2_.and was incubated at RT for 5 min, AlCl_3_ (0.15 mL, 10%) was then added. After a further incubation for 6 min at RT, the reaction mixture was treated with 0.5 mL of 1 mM NaOH. Finally, the reaction mixture was diluted with 275 μL of DDW. The reaction mixture was incubated at RT for 20 min and the absorbance maxima was measured at 510 nm. The total proanthocyanidin content (TPrC) was determined as per the protocol previously reported by Vuong *et al.* [13]. To 0.5 mL of plant extract 3 mL vanillin (4%) was added followed by 1.5 mL of conc. HCl and incubated for 15 min at RT. The absorbance was measured at 500 nm. Total proanthocyanidin content was expressed as cathechin equivalent.

### 2.4. In vitro Antioxidant Properties of the Extracts

#### 2.4.1. Free Radical Scavenging Activity (DPPH Method)

The antioxidant activity of the *B. ramiflora* methanolic leaf extract (BME) and standard were assessed on the basis of the radical scavenging effect of the stable DPPH free radical as per the modified protocol by Goyal *et al.* [11]. The DPPH solution (0.006% w/v) was prepared in 95% methanol. Freshly prepared DPPH solution was taken in a test tube and extract was added in different alliquots (100–1000 μg) and the final volume was made up to 2 mL. The discoloration of the reaction mixture was measured at 517 nm for 30 min of incubation in the dark. The experiment was performed at least in triplicate. Ascorbic acid was used as a reference standard and dissolved in DDW to make the stock solution with the same concentration (1 mg/mL). Methanol (95%) was used as blank. Percentage scavenging of the DPPH free radical was measured using the equation:

(1) $$\text{Percentage}\;\text{scavenging}\;=\;A_{0}-A_{1}/A_{1}\;\times \;100$$

where *A*_0_ = Absorbance of the control and *A*_1_ = Absorbance in the presence of the sample. The actual decrease in absorption induced by the test compounds was compared with the positive controls. The IC50 value was calculated using the dose inhibition curve.

#### 2.4.2. Nitric Oxide Scavenging Activity

NO generated from sodium nitroprusside was measured by the Greiss reaction [14] as per Goyal *et al.* [15]. The absorbance of the chromophore formed due to diazotization of sulphanilamide with nitrite and subsequent coupling with napthylethylene diamine was read at 540 nm in a UV-visible spectrophotometer. Percentage scavenging was measured as per Equation (1).

#### 2.4.3. Determination of Total Antioxidant Capacity by Phosphomolybdenum Assay

The total antioxidant capacity of BME was assessed by the phosphomolybdenum method [16]. To 0.3 mL of extract (1 mg/mL), 3 mL reagent solution (28 mM sodium phosphate, 6 M sulfuric acid and 4 mM ammonium molybdate) was added and incubated at 95 °C for 90 min. Then the absorbance of the solution was measured at 695 nm against a blank. The antioxidant capacity of extract was evaluated as equivalents of ascorbic acid.

### 2.5. Quantitative Analysis of Antioxidant Compounds Using High-Performance Liquid Chromatography (HPLC)

#### 2.5.1. Preparation of Standard and Sample Solutions for HPLC

Stock solution of rosmarinic acid was prepared at a concentration of 25 μg/mL just prior to use and used as reference standard. BME was dissolved in HPLC grade methanol to get the desired concentration and used as sample. Prior to injection both the sample and standard were filtered through a 0.22 μm millipore filter.

#### 2.5.2. Chromatographic Conditions for HPLC

The HPLC system (Waters, Singapore) consisted of photodiode array detector (W2998), dual pump system (515-waters), temperature control module II (TC2-waters), pump control module (PC2-waters), system controller (EMOAA01712) and a reverse phase HPLC analytical column-waters Spherisorb C8, 4.6 × 100 mm, 5 μm particle size. The extract and standard were passed through a 0.22 μm filter (Merck Millipore, Darmstadt, Germany) before injection into a reverse phase NOVA-PAK C18 (Waters, Singapore) column (4.6 × 100 mm, particle size 4 μm) at ambient temperature (20 °C). A Waters 515 system controller coupled with a photodiode array detector (Waters 2998 series) was used. The mobile phase was Acetonitrile (A) and water containing 0.1% Phosphoric acid (B). The gradient was as follows: 0 min, 5% A; 10 min, 15% A; 30 min, 25% A; 35 min, 30% A; 50 min, 55% A; 55 min, 90% A; 57 min, 100% A and then held for 10 min before returning to the initial conditions. The flow rate was adjusted to 1.0 mL/min, sample run time was 30 min and the detector was set at 320 nm at 1.2 nm resolution with the mobile phase methanol: water (50:50 v/v, isocratic) [17]. Active constituent in the sample was identified by comparison of the retention time with a standard. Data was analyzed using Empower software (Waters, Singapore).

### 2.6. In vivo Assay

#### 2.6.1. Animals

Male *Wistar albino* rats (140–160 g) were housed under standard laboratory conditions of light and dark cycles of 7:00 am to 7:00 pm, temperature of 25 °C ± 2 °C and 68% ± 1% relative humidity. The animals were provided standard rat pellet (Lipton India Ltd., Bangalore, India) and tap water *ad libitum*. The study protocol was approved by Maharani Lakshmi Ammanni College Ethical Committee, clearance from ethical committee (1368/ac/10/CPCSEA), Bangalore.

#### 2.6.2. Acute Toxicity Test

Swiss albino mice (25–30 g) of both sexes were divided into six groups of 10 each. Animals were fasted overnight and were free to access water preceding the experiment. BME was administered to each group at different dose levels of (0.5, 1.0, 1.5, 2.0, 2.5, 3.0 g/kg BW/mL). The mice were critically observed for 24 h, mortality was recorded and LD_50_ (Median lethal dose) was determined as per author’s previous studies [18].

#### 2.6.3. Experimental Groups

Rats were randomly divided into five groups of six rats each. NL: Normal (1 mL/kg *p.o* dimethyl sulphoxide, DMSO), IC: carrageenan injected control; IC + low dosage of BME (LBME): control rats treated with 100 mg/mL of *B. ramiflora* methanolic leaf extract; IC + high dosage of BME (HBME): control rats treated with 200 mg/mL of BME; IC + diclofenac: control rats treated with diclofenac (10 mg/kg) (Sigma Chemical Co., St. Louis, MO, USA) dissolved in DMSO.

### 2.7. Anti-Inflammatory Activity

The carrageenan-induced rat paw oedema test [19] has been used as an experimental model for testing the anti-inflammatory activity as per previous reports [20]. One hour after the oral administration of LBME and HBME or diclofenac, carrageenan-saline solution (0.5%, w/v) and saline were injected in a volume of 0.1 mL into the plantar surface of the right and left hind paw, respectively. The left paw served as the control (non-inflamed) paw. The experimental animals were observed for 0 h, 1 h, 2 h and 4 h; the paw volume was measured using a plethysmometer (PanLab, Barcelona, Spain). The anti-inflammatory effect was calculated using Equation (2):

(2) Anti-inflammatory effect (%) = k − e/k × 100

where *k* is the difference in the paw weight in the control group, and *e* is the difference in the paw weight in the treatment group. Blood samples were collected by retro-orbital puncture from all rats and serum was separated for further biochemical estimations.

### 2.8. Measurement of IL-1β, TNF-α Level

The serum concentration of interleukin 1 beta (IL-1β) and tumor necrosis factor alpha (TNF-α) (Endogen, Woburn, MA, USA) were measured using a commercial enzyme-linked immunosorbent assay (ELISA) kit method (Biosource, San Diego, CA, USA) [21].

### 2.9. Statistical Analysis

All the experiments were repeated six times and expressed as mean ± standard error of means (SEM) and statistically analyzed by two-way analysis of variance followed by Tukey’s multiple range using Graph pad prism. *p* < 0.05 and *p* < 0.01 was considered to be statistically significant.

## 3. Results and Discussion

### 3.1. Yield of the Extract

Total yield of the extract was found to be 8.4%.

### 3.2. Determination of Biochemical Constituents

The total phenol, flavonoid and proanthocyanidin content of the BME were found to be 79.06 ± 0.03 mg GAE/g, 28.80 ± 0.01 mg QE/g and 29.42 ± 0.01 μg cathechin/g respectively. The methanolic fraction of *B. ramiflora* was found to be rich in phenols, flavonoid and proanthocyanidin as compared to other polar and non-polar fractions. The higher polyphenolic content of BME might be due to the potential of methanol to release the bound polyphenols present in the cell wall of the plants. Thus, methanol proves to be a suitable solvent for extraction of the plant [22,23].

### 3.3. In Vitro Antioxidant Properties of the Extracts

#### 3.3.1. Free Radical Scavenging Activity (DPPH Method)

**Figure 1:**
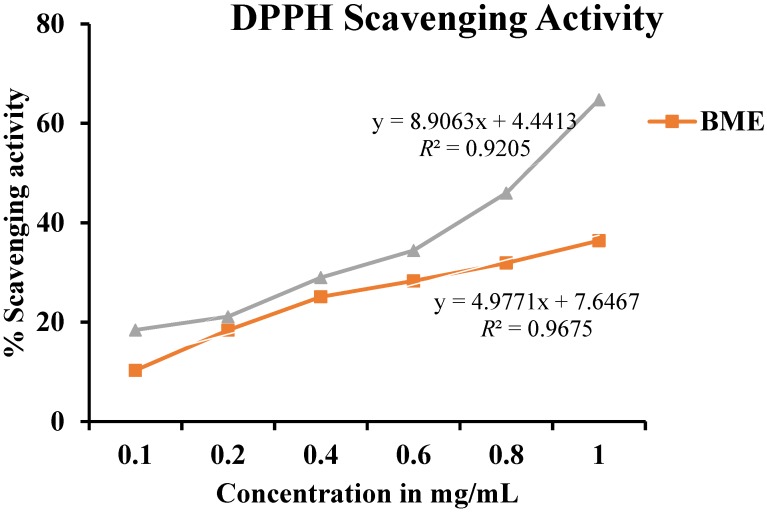
2,2-Diphenyl-1-picryl-hydrazyl (DPPH) scavenging activity of methanolic extract of *Baccaurea ramiflora* leaf.

BME showed radical scavenging activity as measured by DPPH, across concentrations ranging from 0.1 to 1.0 mg/mL (Figure 1). The value for maximum % inhibition was noted at a concentration of 1.0 mg/mL with 36.4% inhibition, whereas that of standard (ascorbic acid) was found to be 64.76%. The radical scavenging activity, using DPPH exhibited the highest radical scavenging activity reveals that BME contains powerful inhibitor compounds, which act as potential antioxidants and thus scavenge the DPPH radicals to form stable reduced DPPH molecules [24].

The high DPPH scavenging activities of the methanolic extract have already been reported in comparison to the aqueous and acetone extract by other researchers [22,23].

#### 3.3.2. Nitric Oxide Scavenging Activity

**Figure 2:**
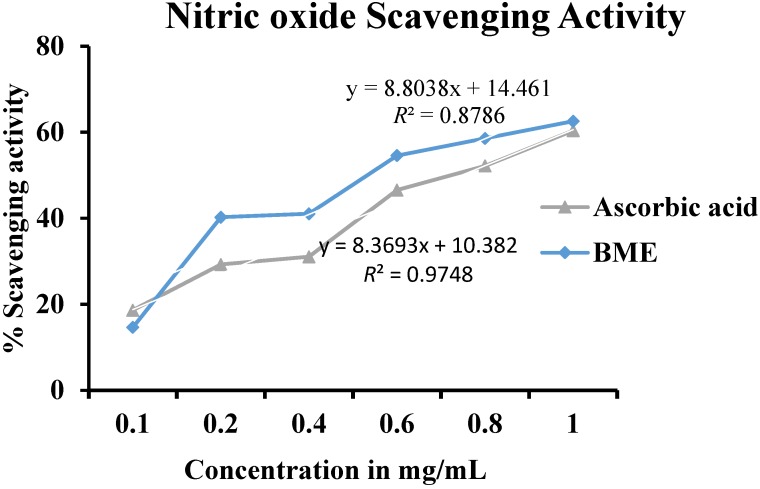
Nitric oxide scavenging activity of methanolic extract of *Baccaurea ramiflora* leaf.

A dose dependent increase in NO scavenging activity was noted in BME (Figure 2). At the concentration of 1.0 mg/mL both BME and the standard ascorbic acid showed comparable NO scavenging ability of 61.33% and 60.32% respectively. Nitric oxide is a powerful intermediary of a number of physiological processes [15]. It is a diffusible free radical that can act as an effective molecule in several biological systems like vasodilation, neuronal messenger, antimicrobial and antitumor activities [25]. The strong NO scavenging activity in a dose dependent manner depicts BME as a potential source of natural antioxidants.

#### 3.3.3. Phosphomolybdenum Assay

This assay deals with the reduction of Mo (VI) to Mo (V) either in the presence of the extract or standard ascorbic acid. BME was more effective in reducing Mo (VI) to Mo (V) (IC_50_ 58.47 ± 1.17 μg/mL) than ascorbic acid (IC_50_ 81.3 ± 4.12 μg/mL), suggesting the presence of effective antioxidants in BME, which was also dose dependent, which increased in a concentration-dependent manner.

### 3.4. HPLC Analysis

**Figure 3:**
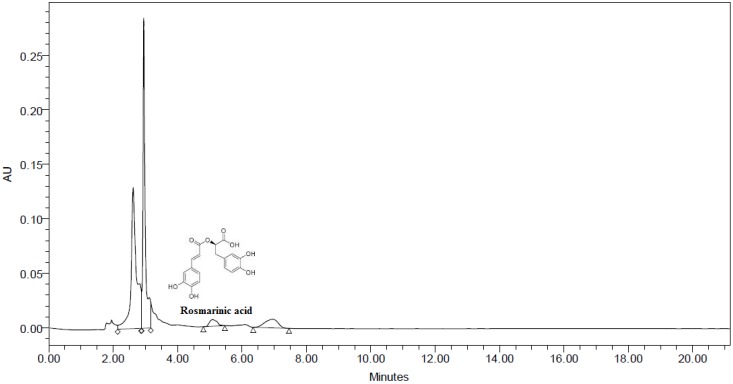
HPLC Chromatogram of methanolic extract of *Baccaurea ramiflora* leaf (BME) showing the presence of rosmarinic acid.

Due to high polyphenolic content and strong antioxidative properties BME was subjected to HPLC. HPLC being simple and easy to use has been found to be an effective technique in identification and quantification of major phenolic compounds present in plants. The chromatogram tested at 320 nm is depicted in Figure 3. The chromatogram shows the presence of rosmarinic acid with retention time of 5.087 when compared to standard rosmarinic acid with RT of 5.084. The content of rosmarinic acid was found to be 0.0008% w/w or 8 mg/kg. This was the first ever study to detect the presence of rosmarinic acid in BME. Rosmarinic acid is an ester of caffeic acid and 3,4-dihydroxyphenyllactic acid which is commonly encountered in species of Boraginaceae and the subfamily Nepetoideae of Lamiaceae along with other higher plants [26].

### 3.5. Assessment of Pharmacological Activity (In Vivo)

There are very few studies that prove pharmacological activities of *B. ramiflora.* The leaf of this plant is known to have hypoglycemic and hypolipidemic properties [8,27]. Hence to add to the pharmacology activity the present study focused on the anti-inflammatory activity of the methanolic fraction of *B. ramiflora leaves*.

#### 3.5.1. Toxicity

BME administration did not exhibit any side effect in survival rate and body weight of the experimental animals. *In vivo* LD_50_ of the BME was estimated to be 1.980 g/kg BW. *In vivo* methods of estimation of toxicity are of critical value in the drug discovery process. They are carried out to evaluate the level to which a chemical entity can cause injurious effects to the cell and to broadly understand the optimum drug concentration to be administered. Determined LD_50_ was used to define the dosage for the study and to the best of our knowledge, this is the first study to report the lethal dose of BME.

#### 3.5.2. Anti-Inflammatory Activity

Carrageenan injection into the rat paw provokes a local, acute inflammation because of arachidonic acid metabolites. The BME extract showed dose dependent inhibition of carrageenan induced edema comparable to the anti-inflammatory drug diclofenac. BME significantly (*p* < 0.05) hindered the incidence of inflammation and a dose dependent reduction of paw edema was seen at 4 h. After 4 h diminution in paw edema by HBME was 62.71%, as compared to edema reduced (71.57%) by the standard drug, diclofenac (10 mg/kg) (Table 1). The BME was found to be highly effective in inhibiting edema. It is evident from our results that phytoconstituents (Rosmarinic acid) would be responsible for the anti-inflammatory activity which might be related to the inhibition of the release or synthesis of cyclooxygenase products. However detailed molecular and chemical level study is required to confirm the same. The results also correlate with our previous studies where phytochemical screening indicates the presence of phenols, flavonoids and terpenoids which might add to the pharmacological activity [1]. Rosmarinic acid is known to possess extraordinary biological anti-inflammatory, anti-mutagen, and anti-microbial activities [28]. The antioxidative potential of rosmarinic acid has been established by various workers [29,30].

**Table 1:** Paw volume variation due to anti-inflammatory effects of *Baccaurea ramiflora* (BME) ^&^.

Experimental Animals	Dosages mg/kg	% Increase 1 h	% Increase 2 h	% Increase 4 h
NL	No treatment	0.24 ± 0.12	0.62 ± 0.18	0.36 ± 0.24
IC	No treatment	2.1 ± 0.47 ^a^	6.2 ± 0.54 ^a^	9.4 ± 1.76 ^a^
IC + LBME	100	9.3 ± 0.65 ^NS^	14.2 ± 2.17 ^a^	29.6 ± 4.62 ^b^
IC + HBME	200	13.7 ± 1.75 ^NS^	31.3 ± 4.13 ^b^	62.71 ± 4.98 ^b^
IC + Diclofenac	10	52.3 ± 6.89 ^b^	60.1 ± 8.90 ^b^	71.57 ± 7.32 ^b^

^&^ Data represented as mean% ± SEM for number of animals = 6. NL: normal; IC: carrageenan injected control; IC + LBME: control rats treated with 100 mg/mL; IC + HBME: control rats treated with 200 mg/mL; IC + Diclofenac: control rats treated with diclofenac. Different subscripts denotes significant group at *p* < 0.05 *vs.* inflammatory group (IC); ^NS^ indicates not significant.

The higher antioxidant activity of BME might be due to the synergistic effect of the presence of rosmarinic acid in conjunction with other phyto-constituents [31]. Edema in rat paw induced by carrageenan is used by and large as an effective inflammatory model for new anti-inflammatory drug design [32]. In the first 2 h (phase I), there is liberation of histamine and serotonin in the rat paw after the carrageenan injection. The complementary system increases the activation and release of kinin, prostaglandin, bradykinin, leukotrienes, and polymorphonuclear cells in the damaged tissue in surroundings cells in phase II [33].

#### 3.5.3. Anti-Inflammatory Effect of BME on Cytokines Levels

Cytokines, small glycoproteins, are produced by the body in response to an antigen, and a mediator for regulating the innate and adaptive immune effects. IL-1β and TNF-α are the major cytokines in inflammation [34]. The cytokine level should be suppressed to reduce the severity of the inflammatory reaction. Table 2 shows that the inflammatory reaction induced by carrageenan significantly uplifts the concentration of IL-1β and TNF-α (*p* < 0.05; *p* < 0.01). BME treatment significantly reduced the production of these cytokines. The anti-inflammatory activity of the standard drug diclofenac sodium, normal saline and two doses of BME extract were compared. In this process, HBME showed a partially significant result compared to the standard drug (*p* < 0.01). Our *in vivo* results were in accordance with a previous report [35]. TNF-α and interleukin-1β levels of experimental animals were significantly lowered with HBME induction. HBME was more effective than LBME and comparable with the standard drug.

Earlier reports also indicated the potential role of phenolic compounds in inflammatory conditions [35,36] and their ability to inhibit polymorphonuclear lipoxygenase, an enzyme involved in inflammatory conditions [37].

**Table 2:** Cytokines levels (interleukin-1β (IL-1β) and tumor necrosis factor-α (TNF-α) of experimental animals (*n* = 6).

Experimental Animals	IL-1β (pg/mg Protein)	TNF-α (ng/μg Protein)
NL	3.3	0.15
IC	10.4	0.58
IC + LBME (100 mg/kg)	6.7 *	0.39 *
IC + HBME (200 mg/kg)	4.4 *	0.21 ***
IC+Diclofenac	4.8 **	0.18 ***

NL: normal; IC: carrageenan induced edema control; IC + LBME: control rats treated with 100 mg/mL; IC + HBME: control rats treated with 200 mg/mL; IC + Diclofenac: control rats treated with Diclofenac. Values are in means ± SEM. * *p* < 0.05 *vs.* inflammatory group; ** *p* < 0.01 *vs.* inflammatory group; *** *p* < 0001 *vs*. inflammatory group.

## 4. Conclusions

This is reputed to be the first report to provide scientific evidence of the biological activities of the traditional use of *Baccaurea ramiflora*. Rosmarinic acid, detected by an HPLC method is thought to inhibit eicosanoids such as prostaglandin biosynthesis which is the end product of the cyclooxygenase pathway. The phytochemical also has the ability to inhibit the infiltration of neutrophils and its degranulation, thereby decreasing the level of arachidonic acid which is responsible for painful sensation. In conclusion this study supports the mechanism involved in the anti-inflammatory and antioxidant activity of *Baccaurea ramiflora*.
